# Impact of botanical oils on polyunsaturated fatty acid metabolism and leukotriene generation in mild asthmatics

**DOI:** 10.1186/1476-511X-12-141

**Published:** 2013-10-02

**Authors:** Jonathan P Arm, Joshua A Boyce, Lin Wang, Heng Chhay, Muhammad Zahid, Vaishali Patil, Usha Govindarajulu, Priscilla Ivester, Kelly L Weaver, Susan Sergeant, Elliot Israel, Floyd H Chilton

**Affiliations:** 1 Division of Rheumatology, Immunology and Allergy, Brigham and Women’s Hospital, 75 Francis St 02115, Boston, MA, USA; 2 Pulmonary Division, Brigham and Women’s Hospital, 75 Francis St 02115, Boston, MA, USA; 3 Center for Clinical Investigation, Brigham and Women’s Hospital, 75 Francis St 02115, Boston, MA, USA; 4 Partners Asthma Center, Brigham and Women’s Hospital, 75 Francis St 02115, Boston, MA, USA; 5 Department of Medicine, Harvard Medical School, 25 Shattuck Street, 02115, Boston, MA, USA; 6 Department of Physiology/Pharmacology, Wake Forest School of Medicine, Medical Center Blvd, 27157, Winston-Salem, NC, USA; 7 Center for Botanical Lipids and Inflammatory Disease Prevention, Wake Forest School of Medicine, Medical Center Blvd, 27157, Winston-Salem, NC, USA; 8 Department of Biochemistry, Wake Forest School of Medicine, Medical Center Blvd, 27157, Winston-Salem, NC, USA; 9 Current address: Novartis International AG, CH-4002, Basel, Switzerland; 10 Current address: Department of Surgery, University of Miami School of Medicine, 1600 NW 10th Ave, 33136, Miami, FL, USA

**Keywords:** Asthma, Gammalinolenic acid, Stearidonic acid, Inflammation, Leukotrienes, Borage oil, Echium oil

## Abstract

**Background:**

Dietary supplementation with botanical oils that contain n-6 and n-3 eighteen carbon chain (18C)-PUFA such as γ linolenic acid (GLA, 18:3n-6), stearidonic acid (SDA, 18:4n-3) and α linolenic acid (ALA, 18:3n-3) have been shown to impact PUFA metabolism, alter inflammatory processes including arachidonic acid (AA) metabolism and improve inflammatory disorders.

**Methods:**

The diet of mild asthmatics patients was supplemented for three weeks with varying doses of two botanical seed oils (borage oil [*Borago officinalis*, BO] and echium seed oil [*Echium plantagineum*; EO]) that contain SDA, ALA and GLA. A three week wash out period followed. The impact of these dietary manipulations was evaluated for several biochemical endpoints, including *in vivo* PUFA metabolism and *ex vivo* leukotriene generation from stimulated leukocytes.

**Results:**

Supplementation with several EO/BO combinations increased circulating 20–22 carbon (20–22C) PUFAs, including eicosapentaenoic acid (EPA), docosapentaenoic acid (DPA), and dihommo-gammalinolenic acid (DGLA), which have been shown to inhibit AA metabolism and inflammation without impacting circulating AA levels. BO/EO combinations also inhibited *ex vivo* leukotriene generation with some combinations attenuating cysteinyl leukotriene generation in stimulated basophils by >50% and in stimulated neutrophils by >35%.

**Conclusions:**

This study shows that dietary supplementation with BO/EO alters 20–22C PUFA levels and attenuates leukotriene production in a manner consistent with a reduction in inflammation.

## Background

Asthma is a complex disease which involves smooth muscle contraction and inflammation that result in narrowing and obstruction of the airway. Arachidonic acid (AA) metabolism via the 5-lipoxygenease pathway to form leukotrienes has been demonstrated to be particularly important to the pathology of asthma. Specifically, the cysteinyl leukotrienes, LTC_4_, LTD_4_, and LTE_4_, act at GPCRs, CysLT_1_R and CysLT_2_R, to elicit their effects, which include contraction of bronchial smooth muscle, vasodilatation, and mucus secretion within the airways [1,2]. Additionally, LTB_4_ is a potent chemoattractant for granulocytes, effector T cells, and monocytes, acting at a specific GPCR, BLT1 [3]. Collectively, leukotrienes have numerous proinflammatory properties, and leukotriene modifying drugs have proven effective in the management of asthma [4] and allergic rhinitis [5].

In addition to pharmacologic modifiers, supplementation of diets with fatty acid-based marine and botanical oil supplements have been demonstrated to reduce the severity of several inflammatory disorders including asthma. One of the primary mechanisms thought to be responsible for their efficacy has been the capacity of n-6 and n-3 polyunsaturated fatty acids to alter AA metabolism to form eicosanoids. For example, dietary supplementation with fish oils that contain eicosapentaenoic acid (EPA; 20:5, n-3) inhibits leukotriene generation, likely through substrate competition of EPA with AA for the action of cytosolic phospholipase A_2_ and 5-lipoxygenase [6].

**Figure 1:**
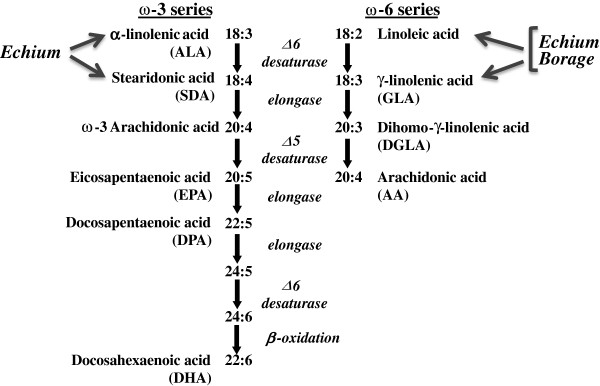
**Pathways for metabolism of n-6 (left) and n-3 (right) PUFAs in humans.** The pathway depicts the synthesis of 20 and 22 carbon PUFAs from the essential, dietary PUFAs, α-linolenic acid (n-3) and linoleic acid (n-6). The PUFAs derived from borage oil (linoleic and gamma-linolenic acids, both n-6) and echium (α-linolenic, n-3; stearidonic, n-3; linoleic and gamma-linolenic acids, both n-6) would be expected to enter the pathways at the indicated points.

In regard to botanical oils, borage seed oils contain the 18 carbon PUFA, gammalinolenic acid (GLA; 18:3, n-6) that is elongated by most tissues and inflammatory cells to dihommo-gammalinolenic acid (DGLA) (Figure 1). Newly-formed DGLA is then incorporated into membrane phospholipids. Like EPA, DGLA has the capacity to compete with AA for eicosanoid production. DGLA is also converted to prostaglandin (PG)H_1_ via cyclooxygenase enzyme(s), which is then converted to PGE_1_. PGE_1_ has been illustrated to have anti-inflammatory effect in both animals and humans [7-9].

In addition, seed oil from another member of the Boraginaceae family, *Echium plantagineum* contains both n-6 and n-3 18C-PUFAs, including a GLA, α-linolenic acid (ALA; 18:3, n-3) and stearidonic acid (SDA; 18:4, n-3). The conversion of ALA to EPA and DHA is poor in humans [10], which is believed to be a result of the inefficiency of the initial rate-limiting step (Δ-6 desaturase, *FADS2* gene) involved in 20–22 carbon PUFA biosynthesis. However SDA is downstream of Δ-6 desaturase and is 4 to 5-fold more efficiently converted to EPA than ALA [11]. Additionally, SDA has been demonstrated to block *in vitro* leukotriene generation and *in vivo* inflammatory processes (reviewed in [11]).

It has been long appreciated that natural products may contain a complex mixture of several active ingredients with synergistic biological effects. As discussed above, botanical seed oils contain several 18C-PUFAs that have the potential to impact diseases that are driven by eicosanoid generation. Several studies have examined the impact of providing individual botanical oils that contain a high proportion of their total fatty acids as a putative PUFA active ingredient; however, little is known regarding the biochemical interactions of adding more than one botanical oil containing several potential PUFA active ingredients. The primary objective of this current study was to examine the biochemical impact of adding PUFAs found in two botanical seed oils on PUFA metabolism and leukotriene generation in mild asthmatic patients.

## Materials and methods

### Subjects

Thirty-seven asthmatic subjects (ages 18–64) were recruited. Written informed consent was obtained from all subjects prior to enrollment. Asthma was diagnosed by the presence of variable airflow obstruction or by a history of treatment for asthma with documented airways hyper-responsiveness to methacholine [12]. Histories (medical and respiratory), a brief physical examination and routine clinical test were used to exclude the presence of significant co-morbid diseases. The inclusion criteria were: 1) male or female 18 years to 65 years of age; 2) asthma with FEV_1_ 50 to 90% of predicted, or personal best; and 3) improvement in FEV_1_ > 12% after administration of a beta-2 agonist. The exclusion criteria were: 1) pregnant or nursing; 2) smoking history of > 10 pack years or active smoking within the previous year; 3) use of asthma treatments that potentially alter leukotriene biosynthesis including theophylline and oral steroids; 4) dietary supplements with fatty acids or other products that may interfere with leukotriene generation; 5) treatment within the previous three months with omalizumab (monoclonal antibody directed against IgE); 6) use of non-steroidal anti-inflammatory drugs in the week prior to any measurements of *ex vivo* leukotriene generation; 7) a history of aspirin-sensitive asthma; 8) significant abnormalities in CBC, differential white cell count, renal function, and liver function, or urinalysis; and 9) any serious co-morbid medical condition.

Subjects meeting the entry criteria were randomized to one of the four study groups based on botanical oil dosing (Table 1). Subjects used rescue albuterol less than twice a week, had nocturnal asthma less than twice a month, and had a forced expiratory volume in one second ≥75% predicted. During the month prior to entry into the study, no subject used leukotriene modifiers, oral or high dose inhaled steroids, or theophylline, and for three months prior to entry into the study no subject used omalizumab, a monoclonal antibody against IgE. The study was approved by the Partners Human Subjects Research Committee. An investigator-initiated IND was obtained from the Food and Drug Administration (IND number 74,110). Because the outcomes in this study were entirely biochemical with no assessment of clinical efficacy, the study was not registered at clinicaltrials.gov.

**Table 1:** Study group compositions and daily dosing scheme for borage and echium seed oils in the four groups of subjects

	**Group 1**	**Group 2**	**Group 3**	**Group 4**
**No. of Subjects**	6	9	11	11
(female)	(3)	(5)	(6)	(7)
**Age (range)**	22–46	18–54	19–58	22–64
**No. of subjects withdrawn**	1	1	1	2
**Echium Oil Dose (g)**	14	7	4	2
No. of daily capsules	14	7	4	2
ALA (g)	4.02	2.01	1.15	0.57
SDA (g)	1.75	0.875	0.5	0.25
GLA (g)	1.54	0.77	0.44	0.22
**Borage Oil Dose (g)**	0	3.9	5.2	5.2
No. of daily capsules	0	3	4	4
ALA (g)	<0.01	<0.01	<0.01	<0.01
SDA (g)	0	0	0	0
GLA (g)	0	0.9	1.2	1.2
**Total**				
No. of daily capsules	14	10	8	6
ALA (g)	4.02	2.01	1.15	0.57
SDA (g)	1.75	0.875	0.5	0.25
GLA (g)	1.54	1.67	1.64	1.44

The number of daily capsules, containing 1 g (EO) or 1.3 g (BO) oil per capsule, and the amount of ALA, SDA and GLA provided by the encapsulated echium and borage oils is shown for each dosing group. Recruitment into Group 1 was stopped after the interim analyses showed that the highest dose of EO was not required to prevent elevations of AA levels in circulating plasma.

### Dietary fatty acids

Capsules containing echium seed oil (1000 mg) or borage seed oil (1300 mg) were supplied by Bioriginal (Saskatoon, SA, Canada). The fatty acid contents of the oils were validated by gas chromatography (Table 2). Since the leaves of *Echium plantagineum* contain pyrrolizidine alkaloid, an independent assay of this toxin was obtained, assessed by high performance TLC, and found it to be less than 4 ng/g of oil (Chemisches Laboratorium Dr. Hermann Ulex Nachf, Hamburg-Norderstedt, Germany).

**Table 2:** Fatty acid profile of botanical oils consumed by asthmatic subjects

	**%*of Total Fatty Acids***	
**Fatty acid**	**Echium**	**Borage**
C16:0	7	6.7
C16:1	0.2	0.2
C18:0	4.1	3.3
C18:1	16.8	10.4
C18:2 n-6	18	24.6
C18:3 n-6	10.8	40.5
C18:3 n-3	28.7	0.1
C18:4 n-3	12.5	0.3
C20:0	0.2	0.3
C20:1	0.7	5.2
C20:1	0	0.3
C22:0	0	0.6
C22:1	0.4	3.8
C24:0	0	0.1
C24:1	0	3.1
Others	0.3	0.5
* Total*	*99.7*	*100*

### Study design

The study goal was to test combinations of Boraginaceae family seed oils derived from *Borago officinalis* (BO) and *Echium plantagineum* L. (EO) for their capacity to impact circulating PUFA levels and inhibit *ex vivo* leukotriene generation. After baseline determinations of plasma fatty acid levels and *ex vivo* leukotriene generation, subjects were randomized to dosing groups (Table 1) ingesting a constant dose of GLA (~1.5 g daily), 0.25 g to 1.75 g SDA and 0.57 g to 4.02 g ALA in divided doses (three times a day) for 3 weeks.

Aside from the addition of supplements, the subject’s diets were not altered before or during the study. The ‘typical’ western diet provides very small quantities of GLA and SDA, and it is difficult to measure these PUFA in circulating or cellular lipids. GLA and SDA are typically only observed when individuals are consuming a GLA- or SDA- containing supplement such as borage, evening primrose, black currant, or echium, and this was an exclusion criterion of the study. ALA (n-3) makes up ~1% of the typical western diet and so the supplement may have some impact on dietary levels. Approximately 7% of energy in the western diet is LA, and thus the quantities of LA in the supplements would have little total impact on overall linoleic acid (n-6) consumption.

Concentrations of GLA provided to human subjects as borage oil alone have been shown to induce increases in circulating AA levels [13]. It was our hypothesis that the presence of the n-3 18C-PUFA SDA in echium oil would prevent such an increase. However, to assure that there were no elevations of circulating AA out of the normal range, an interim analysis was carried out during the early phase of the study. The interim analyses revealed that AA levels remained constant in all groups and consequently, the study was then completed. Blood and urine were collected weekly for assessment of hematological indices, liver and renal function, plasma fatty acids, *ex vivo* leukotriene generation. After three weeks of supplementation, subjects entered a 3 week wash-out phase, in which plasma fatty acids and *ex vivo* leukotriene generation were measured weekly. After dropouts, 6 to 11 individuals completed each arm of the study.

Subjects reported any change in their medical condition at each study visit and used diary cards to record all medical symptoms and to log intake of study oils. Compliance was monitored by medication diaries and counts of returned capsules. These assessments indicated at least 90% compliance by all subjects and plasma fatty acids measurements, in which no outliers were seen, also reflected in a comparable adherence to the supplementation. As the end points of the study were entirely biochemical, the subjects were not blinded (i.e. subjects took different numbers of capsules depending on the group to which they were assigned). The objective of this biochemical study was to determine the impact of potentially bioactive PUFAs in combinations of BO and EO on plasma fatty acid levels and *ex vivo* leukotriene production. All individuals performing the biochemical assays were blinded to the subjects’ study group.

The most common adverse events associated with taking BO/EO combinations were gastrointestinal symptoms, occurring in thirteen subjects, variously reported as gas, constipation, loose stools, and/or abdominal pain or discomfort. With the exception of the 3 subjects who withdrew due to gastrointestinal discomfort (Groups 2, 3 and 4), these symptoms were mild and transient, occurred in the first few days of the study and resolved within 2 to 3 days while still taking BO/EO combinations. Other adverse events included cold-like symptoms (n = 7: Group 1 n = 3; Group 2, n = 2; Group 3, n = 1; Group 4, n = 1 ), pharyngitis (n = 2; one each in Groups 2 and 4), mild symptoms of asthma associated with seasonal allergies (n = 2, one each in Groups 3 and 4), transient wrist pain (n = 1; group 3), and transient itchy rash (n = 1; Group 4). Two other subjects withdrew from the study due to the development of itch and mild asthma flare (Group 4) and unforeseen extension of an out-of-town business trip (Group 1). There were no significant changes in vital signs in any study subjects.

There were no consistent or significant changes in biochemical laboratory parameters or urinalysis during the study (data not shown). However, in four of the individuals who entered the study (2 male, 2 female) there was a decrease in circulating hemoglobin of >1.0 g/dl. Across all study groups there was on average a decrease in circulating hemoglobin of 0.45 g/dl (13.8 to 13.3 g/dl; p < 0.001), which did not vary significantly between study groups (p = 0.09 for differences among groups across time). In three individuals, the value dipped just below the range of normal. There was an accompanying decrease in red cell count from 4.58 to 4.39x10^12^ /liter with no change in mean corpuscular volume. A decrease in hemoglobin concentration was not seen in all subjects. There were no accompanying decreases in white cell count or platelet count.

### Plasma and botanical oil fatty acids analysis

Fatty acid methyl esters were prepared in duplicate plasma samples (100 μl) following a modification of Metcalfe *et al*. [14] and analyzed by gas chromatography as previously described [15]. Encapsulated oils were suitably diluted in hexane and submitted to fatty acid analysis in a manner comparable to that for plasma. Fatty acids in sample were identified based on retention times of authentic fatty acid methyl esters (Supleco, Bellefonte, PA, USA; Cayman Chemicals, Ann Arbor MI, USA; Matreya, Pleasant Gap PA, USA; NuChek Prep, Elysian, MN, USA).

### *Ex vivo* leukotriene generation

*FcϵRI*-*dependent generation of cysteinyl leukotrienes from basophils*. Peripheral blood mononuclear cells (PBMC), typically containing ~3 to 4% basophils, were isolated from 20 mL of blood by density gradient centrifugation over Percoll (GE Healthcare, Piscataway, NJ) as described [16]. As previously described [16], basophils were stimulated in PBMC by cross-linking of the high affinity Fc receptor for IgE (FcϵRI). Under these circumstances, the basophil is the predominant, if not the only, source of cysteinyl leukotrienes. Briefly, cells were primed with 10 μg/L IL-3 on ice and stimulated with 0.01 to 1.0 μg/mL of 15A5, an activating monoclonal antibody to FcϵRI, for 30 min at 37°C. Cysteinyl leukotrienes in the supernatant fluids were stored at −80°C until assayed by immunoassay (Cayman Chemical Company, Ann Arbor, MI or GE Healthcare) for LTC_4_ with 100% cross-reactivity with LTC_5_, 48% and 46% cross-reactivity with LTD_4_ and LTD_5_, respectively, and <10% cross-reactivity with LTE_4_ and LTE_5_.

*A23187*-*stimulated generation of leukotrienes from neutrophil*s. Neutrophils were isolated from heparinized blood by dextran sedimentation, density gradient centrifugation through Ficoll Paque (GE Healthcare, Uppsala, Sweden), and hypotonic lysis of contaminating red cells [17]. Neutrophils (2 × 10^6^) were stimulated (5 min, 37°C) with 0.1 to 10 μmol/L A23187 (Sigma-Aldrich, St Louis, MO) in Hanks buffered salt solution containing 1.25 mmol/L calcium and magnesium salts, 25 mmol/L Hepes, and 1 g/L fatty acid-free bovine serum albumin. Leukotrienes in the supernatants were resolved by reverse-phase HPLC (RP-HPLC) as described [18] and quantified by absorbance at 280 nm (LTB_4_ and all-trans LTB_4_) and 235 nm (5-hydroxyeicosatetraenoic acid, 5-HETE).

### Statistical analysis

Statistical analyses were performed using SAS Version 9.2. We employed a repeated measures mixed model (rmm) analysis for each fatty acid to predict the response in which repeated measures were taken on each subject at each visit (time) and the group and time interactions were modeled along with the main effects. The model utilized a compound symmetry correlation structure since the correlation between pairs of times would be similar and not expected to change across time. For analyses of *ex vivo* leukotriene generation by neutrophils and basophils, we also considered the effect of a range of doses of each stimulus at each visit within the same modeling. We then used adjusted least square mean differences obtained from each rmm model to compare the differences between groups and between times within a single group. All analyses were conducted at a 0.05 level of significance.

## Results

### Plasma fatty acids

**Figure 2:**
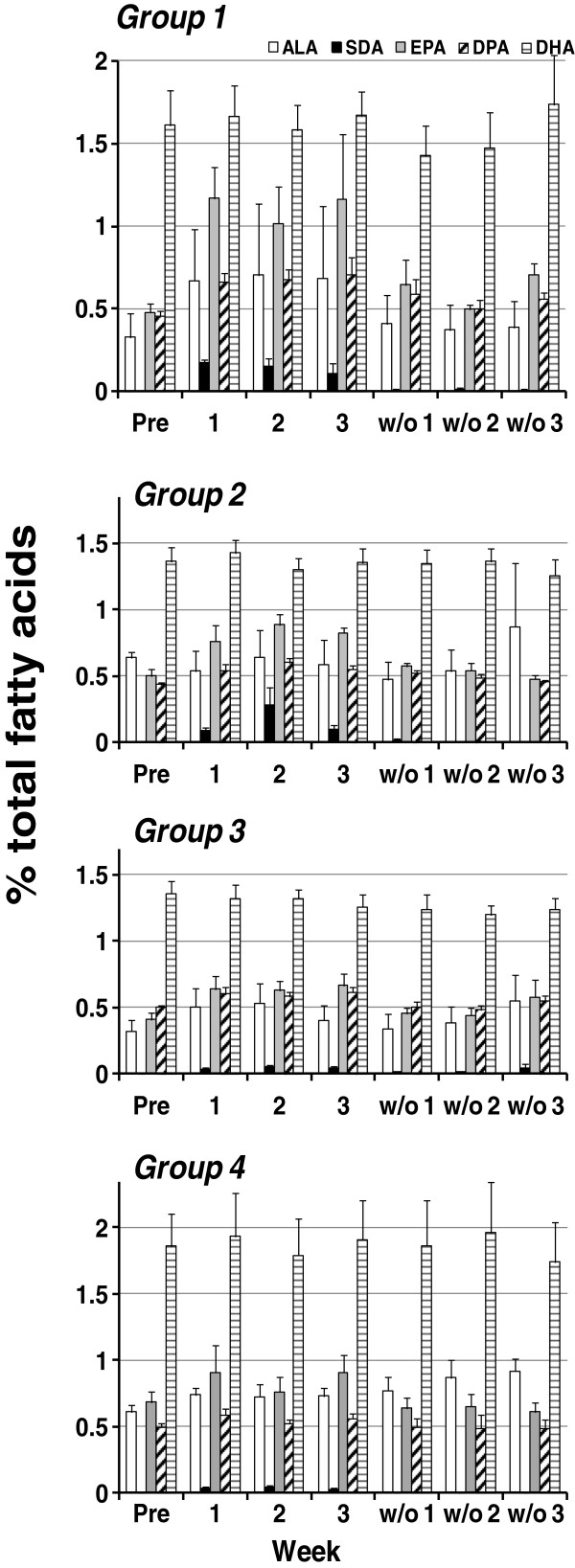
**Effects of dietary supplementation with borage and echium seed oils on concentrations of plasma n-3 fatty acids.** Data are expressed as a percentage of total plasma fatty acids and are shown for ALA (open bar), SDA (black bars), EPA (gray bars), DPA (cross-hatched bars), and DHA (white striped bars). Fatty acid profiles were monitored during the time course of the study beginning at baseline (Pre), during supplementation (weeks 1–3) and during the washout phase (w/o1-3). Data are mean ± SEM for each group. Statistically significant rises in SDA, EPA and DPA were noted over time (p < 0.0001) with significant differences among groups (p = 0.004).

**Figure 3:**
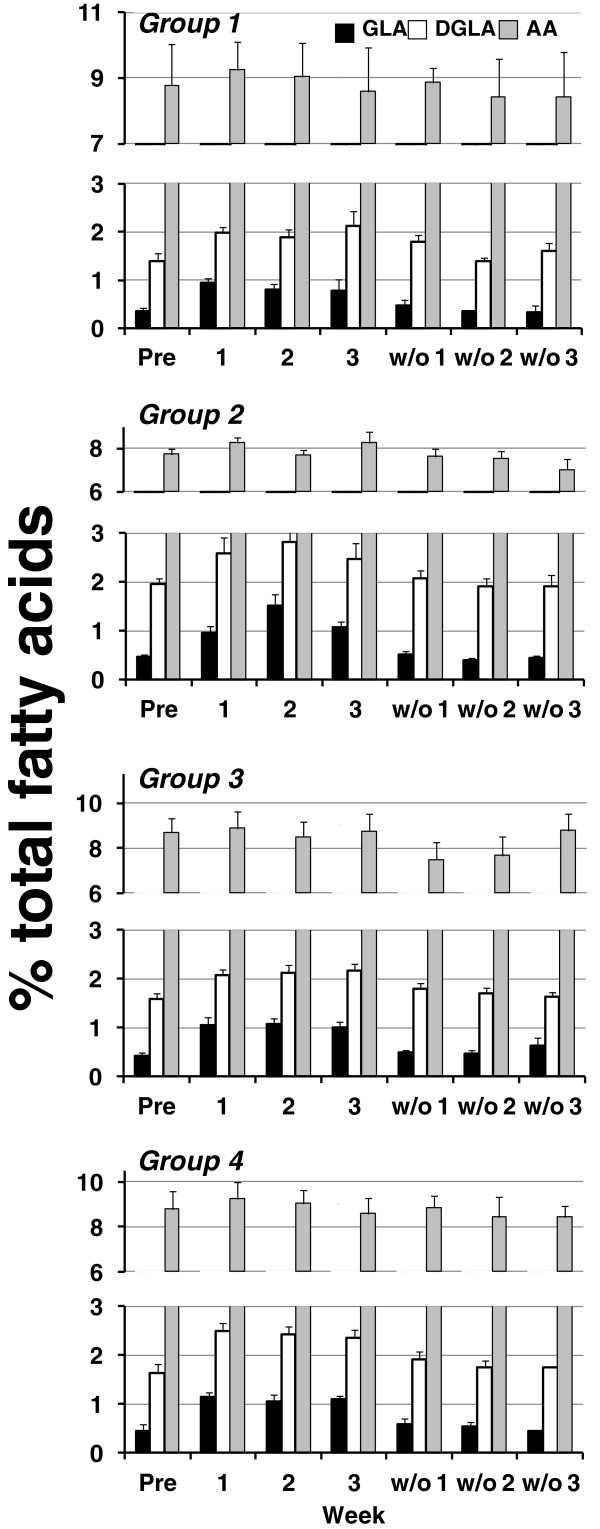
**Effects of dietary supplementation with borage and echium seed oils on concentrations of plasma n-6 fatty acids.** Data are expressed as a percentage of total plasma fatty acids and are shown for GLA (black bars), DGLA (white bars), and AA (gray bars). Fatty acid profiles were monitored during the time course of the study beginning at baseline (Pre), during supplementation (weeks 1–3) and during the washout phase (w/o1-3). Data are mean ± SEM for each group. Statistically significant rises in GLA and DGLA were noted over time (p < 0.0001) with no statistically significant differences among groups.

The effects of dietary supplementation with BO and EO on n-3 and n-6 PUFA levels are provided in Figure 2 and Figure 3, respectively. ALA (18:3n-3), present in echium oil, tended to increase in all groups during the supplementation period (weeks 1–3) and was significantly higher (p < 0.05, Figure 2, white bar) than baseline in Group 1 (wk 3) and Group 3 (wk 2). SDA (18:4n-3) was not detectable in the plasma at baseline but rose significantly during supplementation with BO and EO (p < 0.0001, Figure 2, black bar). As expected, the increase in plasma SDA varied between groups and was most marked in Group 1 in which subjects consumed the largest dose of echium oil (p = 0.004 for differences among groups across time). In each group, the plasma levels of EPA (20:5n-3; Figure 2, gray bar) and its elongation product DPA (22:5n-3; Figure 2, cross-hatched bar) rose significantly (p < 0.0001 for each fatty acid) in a manner that was dependent on the dose of echium oil ingested (p = 0.004 for group by time interactions for each fatty acid). However, there was no rise in circulating plasma DHA (22:6n-3; Figure 2, white striped bar) in any group (p = 0.14). During the washout phase, plasma SDA, EPA and DPA rapidly returned to baseline levels.

With respect to n-6 fatty acids, plasma concentrations of GLA (18:3n-6; Figure 3, black bar) and DGLA (20:3n-6; Figure 3, white bar) rose in each group (p < 0.0001 for each fatty acid) with no significant difference between groups (p = 0.06 and p = 0.63, respectively, for differences among groups across time). Importantly, concentrations of plasma AA (20:4n-6; Figure 3, gray bar) remained constant throughout with no significant change during supplementation with BO and EO in any group (p = 0.39 for effect of time, and p = 0.87 for group by time interactions). During the washout phase, plasma GLA and DGLA rapidly returned to baseline levels.

### *Ex vivo* leukotriene generation

**Figure 4:**
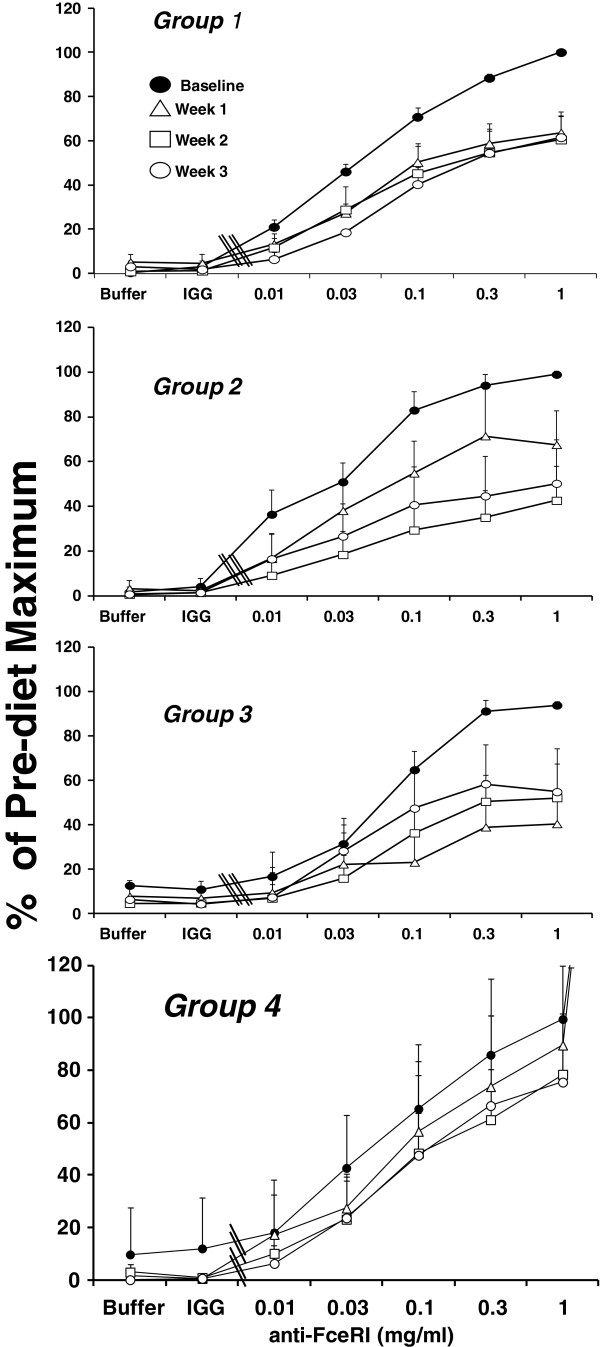
**Dietary supplementation with borage and echium seed oils decreases FcϵRI-dependent cysteinyl leukotriene generation by peripheral blood basophils.** Cysteinyl leukotriene generation is shown prior to (●, closed circles) and one week (∆, open triangles), two weeks (□, open squares), and three weeks (○, open circles) after dietary supplementation with one of four borage and echium seed oil combinations (Groups 1–4, respectively) in response to buffer alone, control IgG1 (1.0 μg/ml, IgG), and 15A5, an activating antibody against FcϵRI (0.01 to 1.0 μg/ml). Due to technical errors, data are not available for one subject in each of groups 1, 3, and 4. In addition, basophils from one subject in each of groups 3 and 4 failed to release leukotrienes upon stimulation, a well described phenomenon due to impaired signaling through *Syk*. Data are expressed as a percentage of maximal cysteinyl leukotriene generation in each subject and are expressed as means ± SEM. Statistically significant suppression of *ex vivo* leukotriene generation was noted (p < 0.0001) with a significant difference between groups (p < 0.0001).

Figure 4 illustrates the effect of dietary supplementation with a constant dose of BO and variable doses of EO on *ex vivo* cysteinyl leukotriene generation from basophils stimulated through FcϵRI. Basophils are the only cell among peripheral blood mononuclear cells that express the fully functional heterotetrameric αβγ_2_ form of FcϵRI and are the principal, if not the only, source of cysteinyl leukotrienes when this population of cells is stimulated through this receptor [16]. We therefore stimulated basophils at ~3 to 4% purity in PBMC with an activating antibody to FcϵRI, before (baseline) and at weekly intervals for three weeks while subjects supplemented their diet with borage and echium seed oils. Because of the marked difference among individuals in absolute quantities of cysteinyl leukotrienes generated by basophils, varying from 1 to 90 ng/10^6^ basophils, data are expressed as a percentage of maximal leukotriene generation per million basophils prior to treatment with BO and EO. Compared to baseline leukotriene generation (Figure 4, closed circles), supplementation with BO and EO resulted in decreased FcϵRI-mediated leukotriene generation. This inhibitory effect was statistically significant in all groups during treatment with BO and EO (p < 0.0001) with a significant difference between study groups (p < 0.0001 for differences among groups across time). The most stable and robust inhibition of leukotriene generation was seen in Group 2 (Figure 4), in which individuals ingested EO/BO combination that provided 1.7 g of GLA, 2.01 g ALA and 0.9 g SDA daily. In this group there was a time-dependent inhibition of cysteinyl leukotriene generation from basophils, stimulated with all concentrations of the antibody to FcϵRI that reached 57% and 50% inhibition of the maximal response by weeks 2 and 3, respectively. A similar inhibition of FcϵRI-dependent cysteinyl leukotriene generation was observed in Group 3 (Figure 4) in which individuals ingested a BO/EO combination that provided 1.6 g of GLA, 1.15 g ALA and 0.5 g SDA daily (n = 8). In Group 3, inhibition was maximal (60%) at week one and cysteinyl leukotriene generation remained suppressed at weeks 2 and 3. Inhibition of FcϵRI-dependent cysteinyl leukotriene generation from peripheral blood basophils was less robust in Groups 1 and 4.

**Figure 5:**
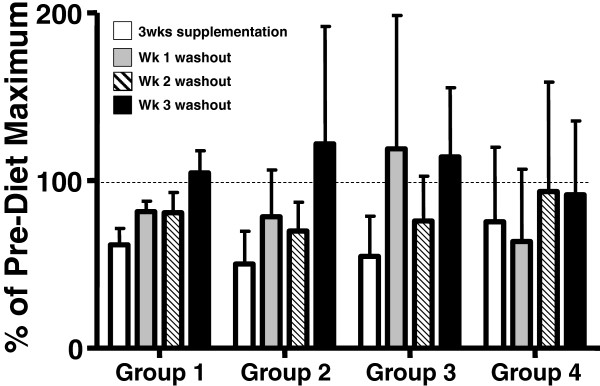
**Cessation of dietary supplementation reverses inhibition of FcϵRI-dependent cysteinyl leukotriene generation by peripheral blood basophils.** After 3 weeks of dietary supplementation (open bars), subjects stopped ingesting borage and echium seed oils. FcϵRI-stimulated basophilic cysteinyl leukotriene generation was measured at one (gray bar), two (cross-hatched bar) and three weeks (solid bar) of washout. Data are shown for maximal cysteinyl leukotriene generation in response to 1.0 μg/ml 15A5. Data are expressed as a percentage of maximal cysteinyl leukotriene generation in each subject and are expressed as means ± SEM.

The inhibitory effect of dietary BO and EO on basophilic leukotriene generation was transient. Figure 5 shows the reversal of *ex vivo* cysteinyl leukotriene generation from basophils (stimulated with a maximum concentration of stimulating antibody) during the washout period after individuals stopped taking capsules of borage and echium oil supplements. Data are expressed as percentage of the maximum cysteinyl leukotriene generation prior to starting supplements. In all groups, there was a return to baseline values within 3 weeks.

**Figure 6:**
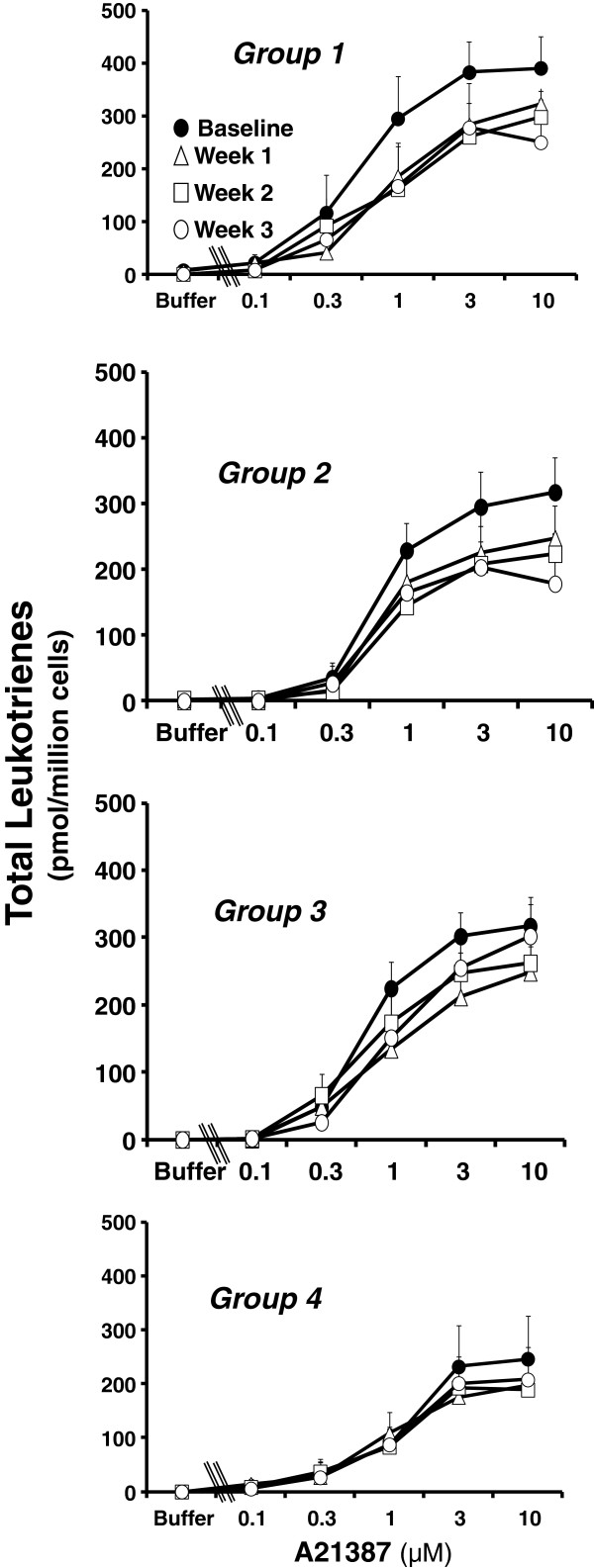
**Dietary supplementation with borage and echium oils decreases A23187-stimulated total leukotriene generation by peripheral blood neutrophil.** Leukotriene generation is shown prior to (●, closed circles) and one week (∆, open triangles), two weeks (□, open squares), and three weeks (○, open circles) after dietary supplementation with one of four borage and echium seed oil combinations (Groups 1–4, respectively) in response to buffer alone and increasing concentrations of the calcium ionophore, A23187 (0.1 to 10 μM). Total leukotriene generation is the sum of LTB_4_, 5-HETE, and, where measurable, all-trans-LTB_4_ isomers. Data are expressed as means ± SEM. Statistically significant suppression of *ex vivo* leukotriene generation was noted (p < 0.0001) with a significant difference in effect between groups (p = 0.02).

A23187 is a calcium ionophore that stimulates leukotriene generation by eliciting calcium flux in a receptor-independent manner. In neutrophils, it provides a robust stimulus for leukotriene generation and allows assessment of the integrity of the whole leukotriene biosynthetic pathway including the terminal product, LTB_4_, and the stable non-enzymatic degradation products of its intermediates, 5-HETE (from 5-hydroperoxyeicosatetraenoic acid) and all-trans-LTB_4_ (from LTA_4_). We therefore examined the effects of dietary supplementation with BO and EO on A23187-stimulated leukotriene generation from neutrophils. The shape and position of the dose response curves for release of LTB_4_ and 5-HETE were similar at baseline, and changes in generation of LTB_4_ and 5-HETE during dietary supplementation with BO and EO were similar (data not shown). Therefore, data are presented as total leukotriene generation; i.e. the sum of 5-HETE, LTB_4_, and all-trans-LTB_4_. A23187 elicited the dose-dependent release of leukotrienes that was apparent at a concentration of 0.3 μM and reached a maximum response at a concentration of 3 to 10 μM A23187 (Figure 6). The quantities of all-trans LTB_4_ diastereoisomers that were generated were small and were not always readily measured by UV absorbance; they are included in the data where possible. Treatment with BO/EO combinations led to a significant inhibition of A23187-stimulated leukotriene generation from neutrophils (p < 0.0001) with a significant difference in effect between groups (p = 0.02 for differences among groups across time). The group-dependent effects of dietary supplementation with BO and EO on A23187-stimulated leukotriene generation from neutrophils were similar to those observed for FcϵRI-dependent cysteinyl leukotriene generation from basophils (Figure 4). The greatest effect was seen in Group 2 (Figure 6), in which maximal total leukotriene generation was inhibited 43% after three weeks of dietary supplementation with BO/EO combinations. The inhibitory effect of the dietary supplementation on neutrophil leukotriene generation declined over time in Group 3 and was less robust in Group 4 (Figure 6). As in the case of basophils, A23187-stimulated leukotriene generation from neutrophils returned to baseline during the 3-week wash-out period (data not shown).

## Discussion

Botanical seed oils from plants such as borage and echium have shown modest efficacy in a number of animal and human inflammation models and disease. These botanicals contain 18C-PUFAs (ALA, SDA and GLA) that can be metabolized into 20–22 carbon PUFAs such as EPA, DHA, DGLA and AA. All of these have been shown to impact eicosanoid generation. However, a better understanding of the *in vivo* biochemistry of potentially bioactive PUFAs found in these botanical seed oils and oil combinations and their capacity to block inflammatory processes including eicosanoid production is needed to enhance the effectiveness of botanical seed oils. The current study utilized various BO/EO combinations to understand these processes.

Supplementation with BO/EO combinations increases plasma levels of n-3 and n-6, 18 carbon and 20–22 carbon PUFAs during the supplementation periods (Figure 2 and Figure 3). Of note, circulating levels of three PUFAs, DGLA, EPA and DPA increased after supplementation. It is likely that DGLA increased as a result of GLA found in both BO and EO. As discussed above, GLA is readily elongated to DGLA in cells and tissues utilizing an enzyme encoded for by a gene known as elongase 5 (*ELOVL5*). Once formed, DGLA is incorporated into inflammatory cells and tissues and competes with AA for the action of cytosolic phospholipase A_2_ and cyclooxygenase to form PGE_1_. Additionally DGLA is converted to a 15-lipoxygenase product, 15-hydroxyeicosatrienoic acid (15 HeTrE) by human mononuclear leukocytes [18]. 15-HETrE has been demonstrated to be a potent blocker of LTB_4_ formation.

Additionally this botanical oil combination increased circulating levels of EPA and DPA This contribution was like due to EO addition since it contains the precursor PUFAs, ALA and SDA. Providing 0.25 g/d to 1.75 g/d of SDA and 0.57 g/d to 4.02 g/d of ALA from EO led to significant and dose-dependent increases in circulating EPA and DPA; plasma EPA concentrations rose more than 2-fold in the group receiving the highest concentration of SDA (Figure 2). This increase in EPA and DPA is likely a result of SDA and not ALA as *in vivo* SDA conversion to EPA is 4–5 fold more efficient than ALA. However, some epidemiological studies suggest that ALA-containing oils (from seed oils such as flax; *Linum usitatissimum L*.) [19] may provide independent protection from cardiovascular disease.

Numerous studies show the biological impact of EPA and DPA. EPA reduces AA metabolism through several mechanisms including decreasing AA mobilization from membrane phospholipids, competition for cylooxygenase and 5-lipoxygenease and reducing the expression of AA metabolizing enzymes and proinflammatory cytokines. Additionally, EPA can serve as a substrate for prostaglandin formation generating “3-series” prostaglandin products including PGD_3_, PGE_3_, PGF_3α_, PGI_3_, and TxA_3_ and “5 series” leukotriene products including LTB_5_ and LTC_5_[20]. Reduced asthma symptoms with n-3 PUFA ingestion have been shown to be related to 5-series leukotriene production [21]. With regard to inflammation, DPA is beginning to receive attention. DPA is converted to 11-hydroxy-7,9,13,16,19-DPA and 14-hydroxy7,10,12,16,19-DPA, which inhibit aggregation of platelets and contain 10-fold greater capacity to elicit endothelial cell migration than EPA, a biological process critical to wound healing [22,23]. There were no changes in plasma levels of DHA, likely reflecting the poor bioconversion of EPA to DHA.

Previous studies have shown that GLA-containing oils such as BO have the potential to increase circulating AA which could enhance inflammation and platelet aggregation through increased thromboxane formation [24]. However, there were no changes in AA levels as a result of BO/EO supplementation. It is possible that the observed increase in EPA resulting from of the botanical combination is a feedback inhibitor of AA production via the ∆5 desaturation step. EPA has been demonstrated to inhibit the *in vivo* and *in vitro* desaturation of DGLA to form AA [25,26]. In any event, the BO/EO combination led to an increase in three 20–22 carbon PUFAs, DGLA, EPA and DPA that have been demonstrated to inhibit AA metabolism and attenuate inflammation without increasing circulating levels of AA.

The final objective of this paper was to determine whether these botanical oil combinations had the capacity to inhibit leukotriene generation from two inflammatory cells, basophils and neutrophils, isolated from subjects with mild asthma who had supplemented their diet with BO/EO. Prior studies of dietary supplementation with GLA have demonstrated a reduction in *ex vivo* leukotriene generation in whole blood or neutrophils stimulated with calcium ionophore A23187 or with zymosan [13,26,27]. Basophils [28], IgE [29], and cysteinyl leukotrienes [30] have been strongly implicated in the pathobiology of asthma. We therefore assessed the effects of BO/EO combinations on the generation of cysteinyl leukotrienes from basophils stimulated through the high affinity IgE receptor, a physiologically relevant stimulus for asthma. Significant inhibition of basophil cysteinyl leukotriene generation was noted within one week of dietary supplementation (Figure 4). Interestingly, the time dependence of this inhibition varied between groups. The least robust inhibition was observed in Group 4, in which subjects received the lowest dose of SDA. Although there is considerable variation in the extent of inhibition of *ex vivo* leukotriene generation among individuals and the groups were relatively small, the between group variation was statistically significant. Furthermore, a comparable variation in supplementation-induced inhibition of leukotriene generation was observed in response to A23187- stimulation of neutrophils (Figure 6). The data therefore suggest that providing SDA in the diet contributed to the extent of inhibition of leukotriene generation, consistent with data showing that dietary supplementation with EPA leads to inhibition of *ex vivo* leukotriene generation [31-33].

In a receptor-independent manner, A23187 robustly stimulates human neutrophils to elicit maximal generation of LTB_4_, the product of leukotriene biosynthesis in neutrophils. Utilization of this stimulus allows an assessment by RP-HPLC of the non-enzymatic degradation products of the proximal intermediates of leukotriene biosynthesis. Dietary supplementation with BO/EO combinations led to a significant inhibition of A23187-stimulated leukotriene generation (Figure 6) that was almost as great as the inhibition of basophil cysteinyl leukotriene generation (Figure 4). We recently reported that dietary supplementation with BO and fish oil led to reduced expression of phosphatidylinositol 3-kinase, a key signaling molecule, in circulating mononuclear cells [15]. It is therefore possible that the inhibition of cysteinyl leukotriene generation that we observed in basophils was due, at least in part, to inhibition of signaling through FcϵRI. However, the inhibition of leukotriene biosynthesis in neutrophils stimulated through A23187, a receptor independent stimulus, argues for a more direct effect of BO and EO on leukotriene biosynthesis.

## Conclusion

Our data demonstrate that ingestion of certain combinations of BO and EO increases circulating levels of both n-6 and n-3, 20–22 carbon PUFAs. Supplementation of human and animal diets with these fatty acids has been shown to inhibit metabolism of AA to pro-inflammatory lipid mediators and ameliorate inflammatory disease models including asthma, arthritis and coronary artery disease [34]. These same combinations do not increase circulating AA levels, as has been observed with BO alone. Importantly, the observed biochemical changes occur concomitantly with a reduced capacity of inflammatory cells from mild asthmatics supplemented with BO/EO combinations to produce leukotrienes that have been illustrated to be important to promoting the asthmatic response. Consequently, an important next step would be to determine whether such BO/EO combinations have the capacity to improve clinical symptoms of asthma.

## Abbreviations

AA: Arachidonic acid; ALA: α linolenic acid; BLT1: B leukotriene receptor 1; BO: Borage oil; CysLT1R: Cysteinyl leukotriene receptor 1; CysLT2R: Cysteinyl leukotriene receptor 2; DGLA: Dihommo-gammalinolenic acid; DHA: Docosahexaenoic acid; DPA: Docosapentaenoic acid; EO: Echium oil; EPA: Eicosapentaenoic acid; GLA: γ linolenic acid; GPCR: G protein coupled receptor; IND: Investigational new drug; LTB4: Leukotriene B_4_; LTC4: Leukotriene C_4_; LTD4: Leukotriene D_4_; LTE4: Leukotriene E_4_; PBMC: Peripheral blood mononuclear cells; SDA: Stearidonic acid.

## Competing interests

FHC is an unpaid consultant for Gene Smart Health and receives no compensation or equity in this role. This information has been disclosed to WFUHS and outside sponsors, as appropriate, and is institutionally managed. All other authors declare no competing interest.

## Authors’ contributions

JPA and FHC designed the study and protocols, oversaw the conduct of the study and wrote the manuscript; EI participated in the conduct of the study; LW and HC performed *ex vivo* experiments and sample analyses; PI and KLW analyzed fatty acids and biomarkers; MZ and VP were study coordinators; UG performed statistical analyses; JAB and SS helped with data analysis and interpretation and manuscript preparation. All authors read and approved the final manuscript.
